# Supplementary material for: Functional Deficits of 5×FAD Neural Stem Cells Are Ameliorated by Glutathione Peroxidase 4
Source: Cells. 2022 May 28;11(11):1770. doi: 10.3390/cells11111770 (PMC9179411; doi:10.3390/cells11111770)
Supplement: Supplementary file 1 [file cells-11-01770-s001.zip › cells-1696694-supplementary.pdf]

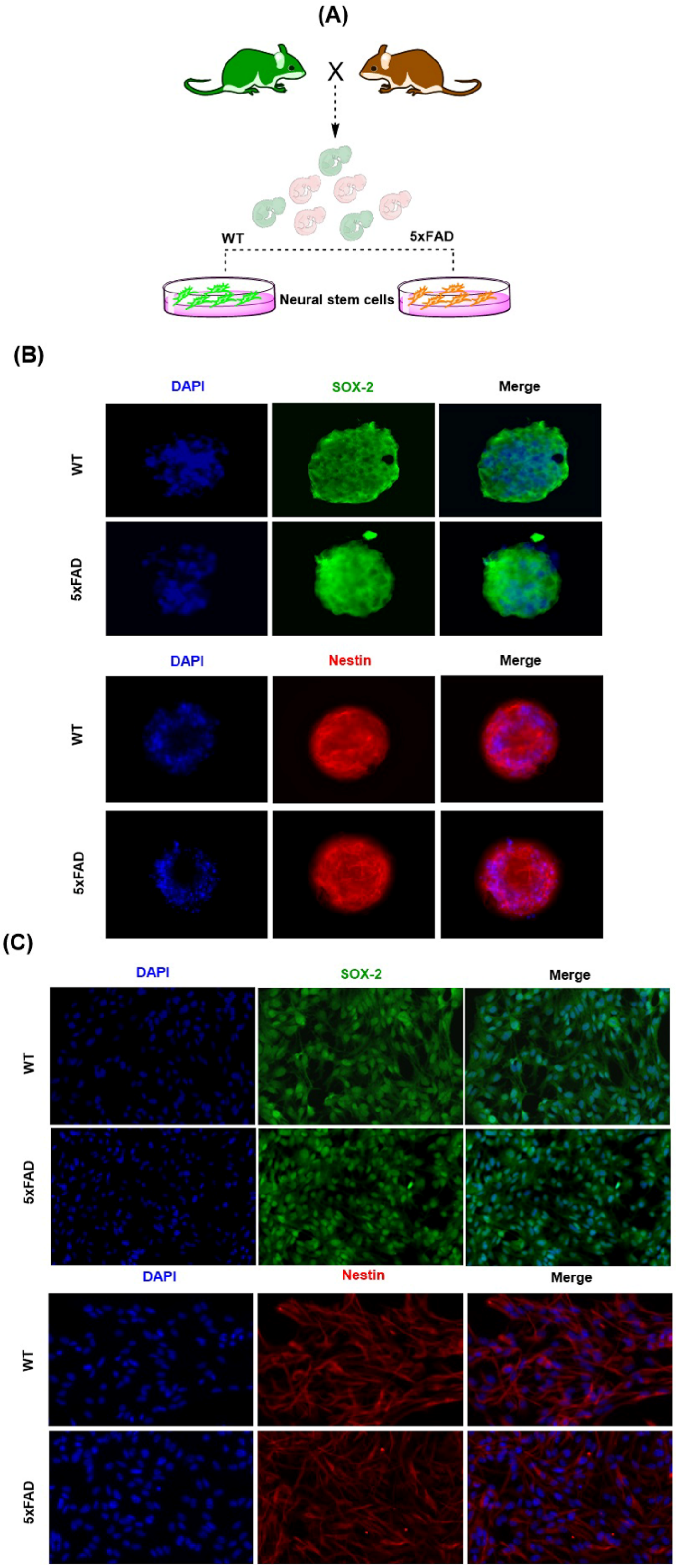
(A)
X
WT
5xFAD
Neural stem cells
(B)
DAPI
SOX-2
Merge
WT
5xFAD
DAPI
Nestin
Merge
WT
5xFAD
(C)
DAPI
SOX-2
Merge
WT
5xFAD
DAPI
Nestin
Merge
WT
5xFAD

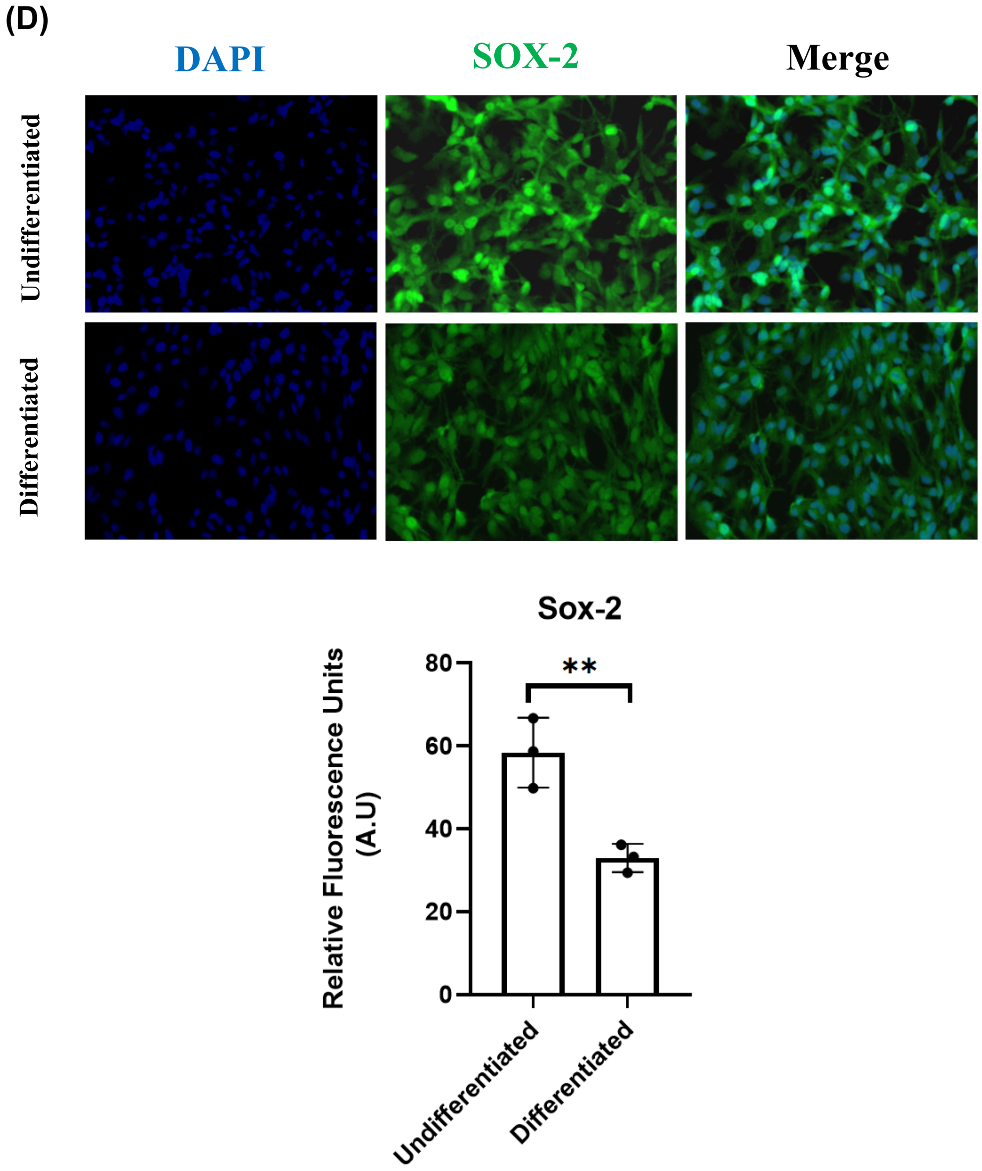

Figure S1. Characterization of NSCs (A). Representative culturing strategy for NSCs from pups **(B).** Representative images of Sox-2 and Nestin (NSC markers) positive staining in neurospheres from WT and 5xFAD groups. **(C).** Representative images of monolayers from WT and 5xFAD groups indicating expression of Sox-2 and Nestin upon differentiation. **(D).** Expression levels of Sox-2 in Undifferentiated and RA differentiated NSCs . **: $p < 0.01$.

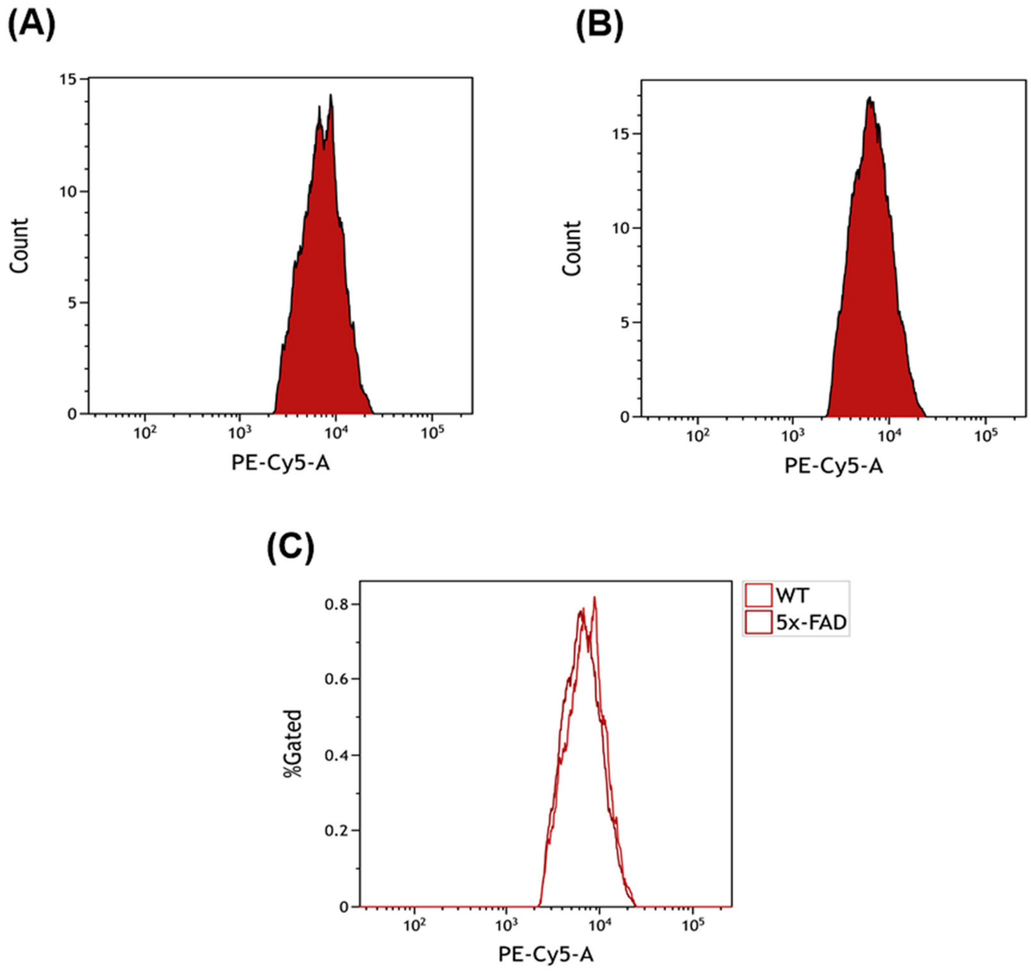

Figure S2. Cell proliferation potential in NSCs: (A) Flow cytometry quantification representing the Edu 647 intensity (cell proliferation indicator) in WT NSCs**, (B)** 5xFAD and **(C)** Merge**.** The data indicates no significant difference in proliferation potential between the groups.

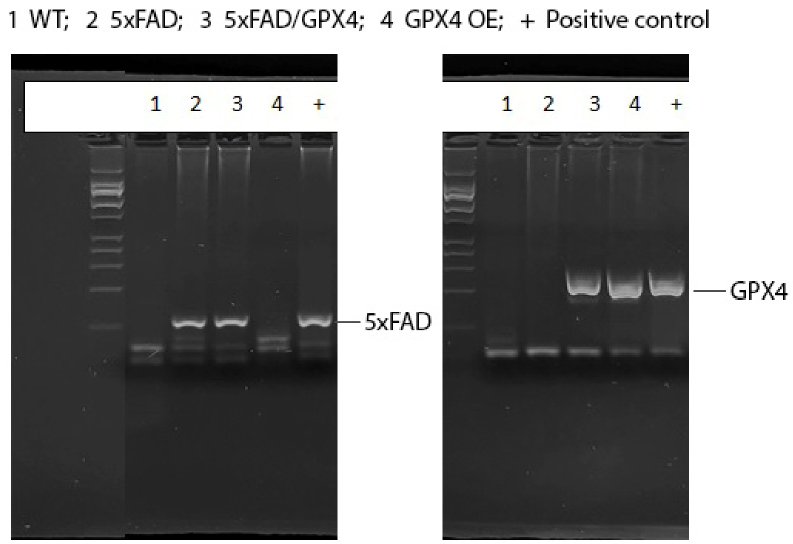

Figure S3. Genotyping profile of NSCs: Representative image of DNA gel indicating the specific bands that differentiate four different genotypes (WT and 5xFAD, 5xFAD/GPX4 and GPX4 OE) of NSCs along with positive controls.

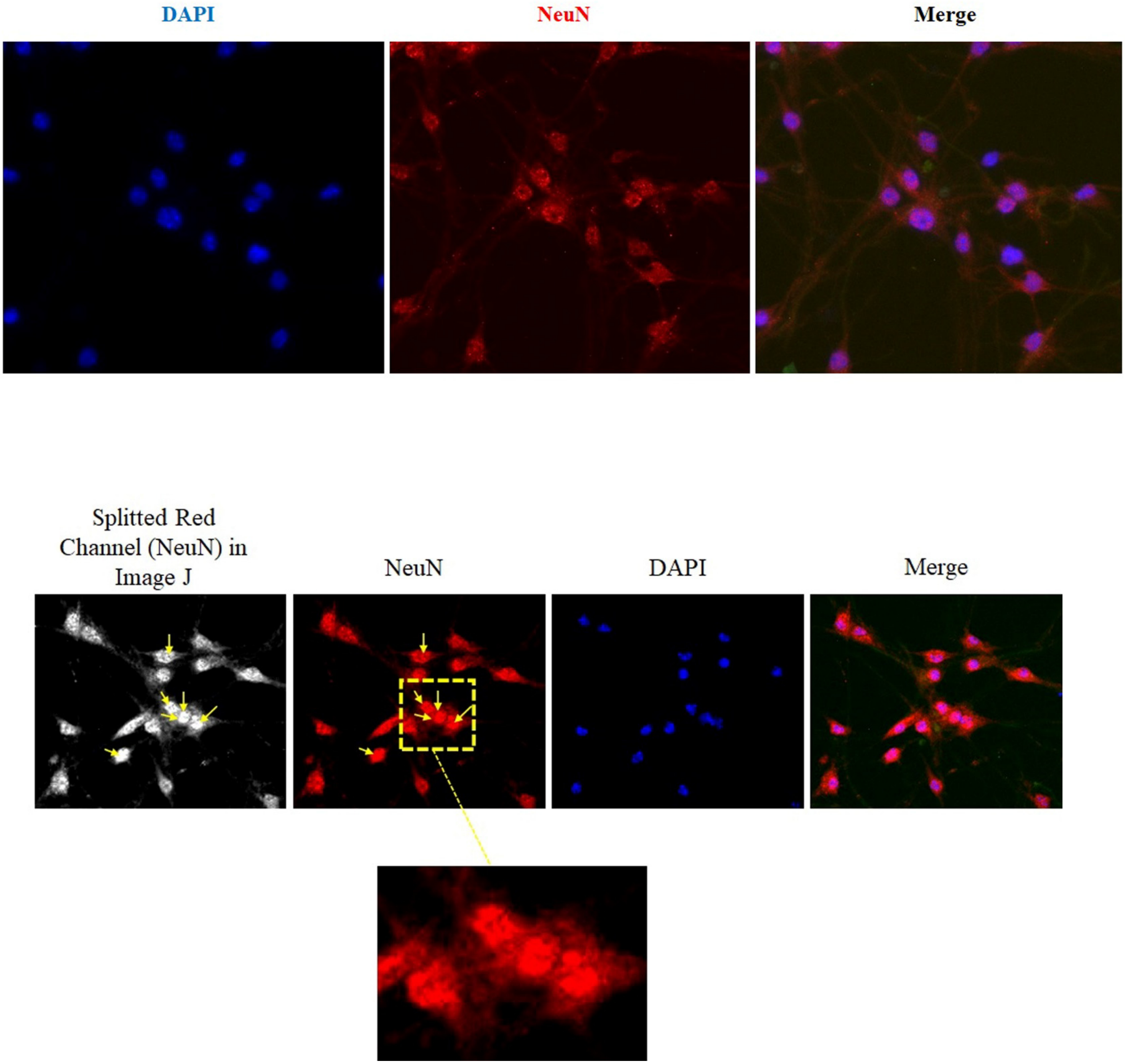

Figure S4: Images of NeuN staining in differentiated WT NSCs cultures
